# sCCIgen: a high-fidelity spatially resolved transcriptomics data simulator for cell–cell interaction studies

**DOI:** 10.1186/s13059-025-03762-9

**Published:** 2025-10-15

**Authors:** Xiaoyu Song, Joselyn C. Chavez-Fuentes, Weiping Ma, Weijia Fu, Sujung Crystal Shin, Pei Wang, Guo-Cheng Yuan

**Affiliations:** 1 Centre for Quantitative Medicine, Duke-NUS Medical School, Singapore, Singapore; 2 Department of Genetics and Genomic Sciences, Icahn School of Medicine at Mount Sinai, New York, NY USA; 3 Institute for Health Care Delivery Science, Department of Population Health Science and Policy, Icahn School of Medicine at Mount Sinai, New York, NY USA; 4 Tisch Cancer Institute, Icahn School of Medicine at Mount Sinai, New York, NY USA

**Keywords:** Data simulator, Spatially resolved transcriptomics, Cell–cell interaction

## Abstract

Spatially resolved transcriptomics (SRT) facilitates the study of cell–cell interactions within native tissue environments. To support method development and benchmarking, we introduce sCCIgen, a real-data-based simulator that generates high-fidelity synthetic SRT data with known interaction features. sCCIgen preserves transcriptomic and spatial characteristics and provides key interaction features, including cell colocalization, spatial dependence of gene expression, and gene–gene interactions between neighboring cells. It supports input from SRT data, single-cell expression data alone, and unpaired expression and spatial data. sCCIgen is interactive, user-friendly, reproducible, and well-documented for studying cellular interactions and spatial biology.

## Background

Understanding cell–cell interactions (CCIs) is essential for unraveling the complexities of biological systems and advancing medical science. CCIs guide cells to form tissues and organs in a precise and coordinated manner. In the immune system, their interactions enable the efficient detection and response to pathogens [1]. Aberrant interactions are often at the heart of diseases. For example, disrupted communication between neurons leads to cognitive decline and memory loss in Alzheimer’s disease [2], while abnormal interactions among cancerous and immune cells facilitate tumor growth and metastasis [3]. Treatments targeting these cellular interactions, such as the PD-1/PDL-1 antibodies targeting the tumor immune escape [4], have demonstrated remarkable effectiveness in clinical settings. Therefore, studying CCIs is fundamental, not only for understanding biological intricacies but also for driving innovations in health and medicine.

Spatially resolved transcriptomics (SRT) technologies serve as a valuable tool for exploring CCIs by enabling researchers to examine the gene expression profiles of cells within the native tissue context [5, 6]. By preserving spatial information, SRT facilitates the study of how the physical distribution of cells relates to their interactions. Various computational tools have been developed to analyze these interactions: some focus on analyzing the spatial colocalization of cells on tissue slides [7]; others explore the association between gene expression levels and the cell-to-cell distances [8, 9]; yet additional tools examine the co-expression of genes, such as from ligand-receptor pairs, in nearby cells [10–14].

Understanding the performance of these rapidly evolving SRT technologies and computational tools is critical for their effective applications. However, this task is challenging due to the lack of a standardized reference dataset with known ground truth. Simulations can offer a potential solution, but existing SRT data simulators, such as SRTsim [15] and scDesign3 [16], have notable limitations (Table 1). First, SRT data is scarce, especially for certain tissues (e.g., human brain) and conditions (e.g., rare disease), yet all existing simulators require SRT datasets for real-data-based simulation. In the meantime, expression data from single-cell and single-nucleus RNA sequencing (scRNAseq/snRNAseq) are more readily available but are not utilized in SRT simulation. Likewise, spatial maps of the cells from alternative sources, such as other conditions or technologies, are increasingly available. These valuable spatial resources are not leveraged to enhance SRT data simulation. Second, existing simulators inadequately capture complex CCIs. Both SRT-specific simulators, like SRTsim [15], and general simulators allowing spatial features like scDesign3 [16] are not designed to effectively capture complex CCIs in generating spatial and transcriptomic data. Third, existing methods mainly focus on reproducing reference SRT data instead of generating de novo patterns, thereby limiting the general applicability. Evaluating beyond the limited patterns captured in existing data is crucial, as tissues, such as tumors, can be heterogeneous and exhibit distinct patterns. Therefore, there is a pressing need to develop an additional SRT data simulator that incorporates abundant data, provides CCIs with ground truth, and allows de novo pattern generation. Such a simulator would significantly advance the development and testing of SRT computational tools and analysis pipelines.

**Table 1:** The comparison of sCCIgen with the existing SRT simulators

		**scDesign3**	**SRTsim**	**sCCIgen**
**Input**	SRT	√	√	√
	***scRNAseq/snRNAseq***			√
	***Unpaired cell-level expression and spatial data***			√
**Spatial**	Estimate window		√	√
	Model input cell spatial distribution	√	√	√
	Mimic real cell density and nonoverlapness			√
	***Allow CCI to affect cell distribution***			√
**Transcriptomics**	Allow spatial (regional) variation	√	√	√
	Model gene–gene correlation	√		√
	***Allow CCI to affect expression—dependent on distributions and expression of other genes of neighboring cells***			√

To address this gap, we introduce sCCIgen, a *s*patially resolved transcriptomics *C*ell–*C*ell *I*nteraction *gen*erator, to simulate high-fidelity SRT data from various spatial and expression references with a focus on CCIs. Similar to the aforementioned existing methods [15, 16], sCCIgen utilizes real data for simulation; however, it presents two major differences. First, sCCIgen uniquely simulates a wide range of CCI patterns, including cell colocalization, spatial dependence of gene expression, and gene–gene interactions between neighboring cells. Second, sCCIgen accommodates reference data from various sources, including single-cell SRT (e.g., SeqFISH +, MERFISH, Xenium), single-cell transcriptomics data (e.g., scRNAseq/snRNAseq), and transcriptomics data combined with spatial maps from alternative sources (e.g., other tissue samples or technologies). sCCIgen faithfully reproduces several key structures in real data and also supports the generation of de novo patterns, making it especially valuable when reference data are limited.

## Results

### Overview of sCCIgen

**Fig. 1:**
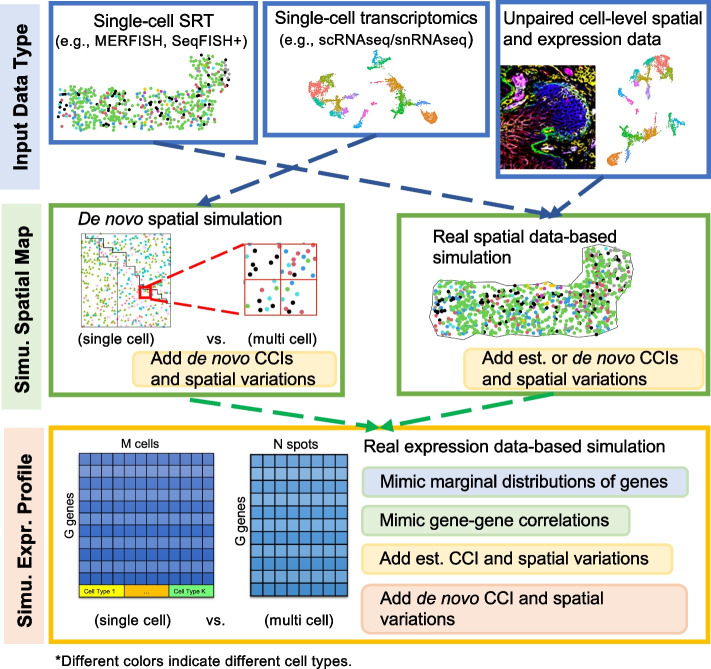
Overview of sCCIgen. sCCIgen operates as a real-data-based simulator, accepting input from single-cell SRT, single-cell transcriptomics (e.g., scRNAseq/snRNAseq), and single-cell transcriptomics with spatial information from an alternative source (e.g., different samples, conditions, and technologies). It first simulates spatial maps for cells and subsequently generates the expression profile for each cell. The resulting output can be in either single-cell or multi-cell resolution

sCCIgen contains the simulation of two major components, the spatial map and gene expression profile (Fig. 1; Methods). To maximally utilize available information, sCCIgen can be used in three different scenarios: (1) paired expression and spatial information such as from a SRT dataset, (2) expression information alone such as from scRNAseq/snRNAseq, and (3) unpaired expression and spatial information.

In the first and last scenarios, sCCIgen extracts the spatial information from the reference spatial data, and then uses such information for data simulation (Methods). Briefly, it first estimates the spatial window of the harbored cells, regardless of the slide shape, for both the entire data and separate regions. Then, within the spatial window, it models the spatial distribution of the cells as a function of their spatial coordinates, stratified by cell types. It can also estimate and identify the cell–cell colocalization (e.g., attraction/inhibition) patterns by cell type pairs. With these spatial models, sCCIgen then generates a spatial map by simulating the spatial coordinates of new cells, allowing them to maintain the estimated spatial and CCI patterns. It also allows users to add de novo CCIs and regional variations on the spatial maps to mimic diverse spatial patterns.

In the second scenario, since there is no spatial reference data available, sCCIgen generates spatial patterns de novo based on user-specified parameters. It flexibly allows different numbers and shapes of spatial regions and, within each region, different numbers of cells, cell types, and their composition. It emulates real data by preventing cell overlaps and maintaining balanced cell densities on the slide. It also allows users to introduce CCIs on the spatial maps that attract or inhibit cells of the same or different cell types from appearing in the neighborhood.

The gene expression patterns are simulated by extracting information from the reference cell-level transcriptomics data (Methods), which could be from the reference SRT or scRNAseq/snRNAseq data. If reference spatial and transcriptomic data are paired (Scenario 1), sCCIgen learns parameters at the region, cell type, and cell neighborhood levels, including the sequencing depths, marginal distributions of genes, gene–gene correlations, gene expression changed by CCIs due to proximity of neighboring cells, and expression of gene pairs, such as from Ligands and receptors, changed by CCIs among neighboring cells. These parameters can be directly used to simulate cell-level gene expression profiles based on their spatial coordinates. In Scenario 2, only the reference gene expression data is available, and sCCIgen learns parameters at the cell type level, including the sequencing depths, marginal distributions of genes, and gene–gene correlations. To introduce the de novo CCI patterns affecting gene expression, sCCIgen enables users to customize regions, cell types, genes, and effect sizes that govern the spatial dependence of gene expression and gene–gene interactions between neighboring cells. In Scenario 3, where the spatial distribution of different cell types is from the alternative reference spatial data, the gene expression profile is simulated for these cells based on the unpaired expression reference in the same way as in Scenario 2. As a spatial data simulation tool, sCCIgen can also introduce de novo regional expression variations. When combined with CCIs, sCCIgen generates complex spatial patterns that allow for the evaluation of analytical tools under intricate conditions. Finally, sCCIgen outputs data at single-cell or multi-cell resolutions.

sCCIgen is available through the widely used programming language R. To facilitate its wide usage, sCCIgen supports both a user-friendly Shiny interface and code-based workflow. It is also designed for seamless integration with popular SRT pipelines, including Giotto [17], Seurat [18], and SpatialExperiment [19]. Along with documentation, tutorials, and examples available at https://songxiaoyu.github.io/sCCIgen/, sCCIgen offers pre-simulated datasets so users can examine their analytical strategies without running their customized simulations.

### sCCIgen faithfully reproducesthe spatial and transcriptomic features of diverse reference data types

sCCIgen designs several key features to closely approximate spatial and transcriptomic characteristics of the reference data. To demonstrate its performance, we simulated SRT data using three types of reference inputs, including SRT data with paired expression and spatial profiles, single-cell expression profiles without spatial input, and expression provided together with spatial maps from an alternative source. When SRT data is used as the reference, the performance is also benchmarked with existing methods, SRTsim and scDesign3.

**Fig. 2:**
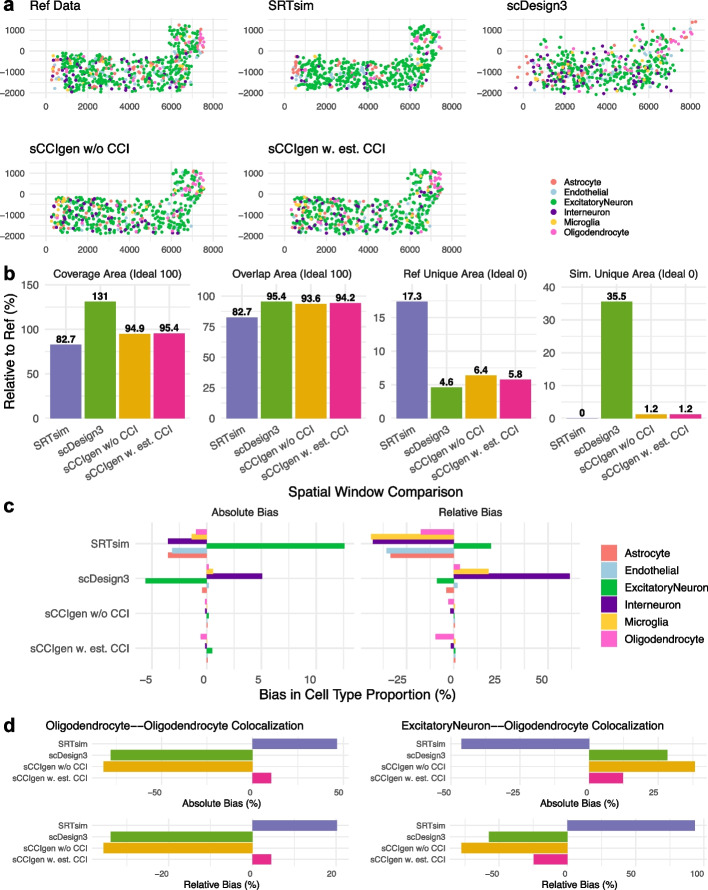
sCCIgen simulated data closely recapitulate the spatial features of reference SRT data from the mouse cortex. **a** Spatial maps of the reference SRT data and simulated data from four methods: SRTsim, scDesign3, sCCIgen without CCI, and sCCIgen with estimated CCI from the reference data. **b** Bar plots comparing spatial coverage metrics, including total coverage (ideal: 100%), overlap with reference (ideal: 100%), reference-only areas (ideal: 0%), and simulation-only areas (ideal: 0%). **c** Bar plots comparing absolute and relative biases in cell type composition from the reference data. **d** Bar plots comparing absolute and relative biases in cell colocalization from the reference data

**Fig. 3:**
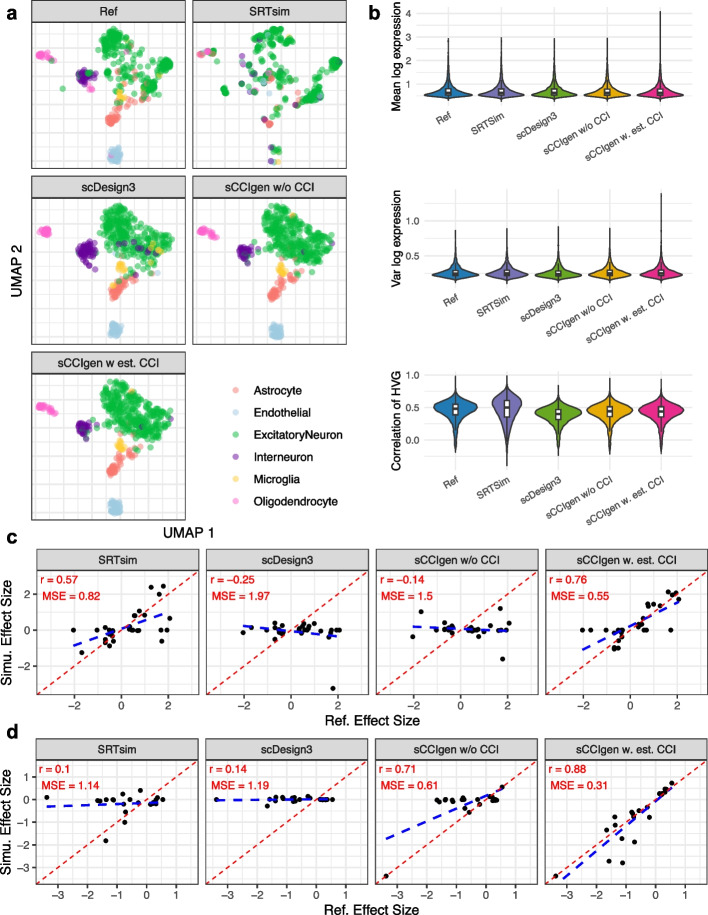
sCCIgen simulated data closely recapitulate the transcriptomic features of reference SRT data from the mouse cortex. **a** UMAP visualization of reference and simulated expression data from four methods: SRTsim, scDesign3, sCCIgen without CCI, and sCCIgen with estimated CCI from the reference. **b** Violin plots comparing key transcriptomic metrics—mean expression, expression variance, and gene–gene correlations among highly variable genes—between the reference and simulated datasets. **c** Scatter plots comparing the correlation and MSE of effect sizes for spatial dependence of gene expression between reference and simulated datasets. **d** Scatter plots comparing the correlation and MSE of effect sizes for gene–gene interactions between neighboring cells between reference and simulated datasets

First, using an SRT dataset as reference, we simulated SRT datasets using SRTsim, scDesign3, sCCIgen without CCI, and sCCIgen with estimated CCI from the reference (Fig. 2: spatial map; Fig. 3: expression profile). The reference data was from a mouse cortex profiled by SeqFISH + technology [20] with 2500 highly variable genes from 511 cells of six cell types, including excitatory neuron, interneuron, astrocyte, endothelial, oligodendrocyte, and microglia. Cell colocalization analysis identified two significant patterns, including cell–cell attraction among oligodendrocytes ($\beta$=2.3) and cell–cell inhibition between oligodendrocytes and excitatory neurons ($\beta$= − 0.49), where the effect size $\beta$ is defined as the enrichment of cell co-occurrence in the neighborhood as compared to randomly perturbed cells (see Methods). sCCIgen with the estimated CCI method leveraged these estimated effect sizes for simulation. As a result, sCCIgen demonstrated notable improvements in the spatial map generated from the existing methods (Fig. 2a), with the upper-right corner enriched with oligodendrocytes and depleted with excitatory neurons when incorporating CCI. Its simulated cells covered 94.9–95.4% of the reference area (Fig. 2b), closely Matching the spatial extent of the original data. In contrast, the SRTsim simulated cells covered only 82.7% of the reference area, while scDesign3 simulated cells covered 131%, largely extending beyond the tissue region. In addition, sCCIgen more accurately reproduced the reference cell-type composition than existing methods (Fig. 2c), with a median relative bias (RB) of 0.4–0.7%, compared to − 34.5% for SRTsim and 2.6% for scDesign3. Here, the RB is defined as (simulated—reference)/reference value. Finally, sCCIgen with estimated CCI exhibited substantially lower biases in oligodendrocyte-oligodendrocyte and excitatory neuron-oligodendrocyte colocalizations relative to the reference data (Fig. 2 d), showing a median RB of − 10%, whereas SRTsim, scDesign3, and sCCIgen without CCI had median RBs of 56.8%, − 45.6%, and − 56.5%, respectively.

sCCIgen also demonstrates a notable improvement of the simulated expression profiles from the existing methods (Fig. 3). UMAP visualization of highly variable genes (Fig. 3a) and the key transcriptomic metrics (Fig. 3b), including mean expression, expression variance, and gene–gene correlations, showed that sCCIgen more closely replicated the reference data than SRTsim and scDesign3. In the meanwhile, the excitatory neurons in the reference data presented some cellular heterogeneity, which was modeled as a single group in all simulators and thus showed homogeneous distributions on UMAP, especially in scDesign3 and sCCIgen. We further evaluated the fidelity of spatial dependence of gene expression by comparing the correlation and mean squared error (MSE) of effect sizes between simulated and reference datasets (Fig. 3c). sCCIgen with estimated CCIs achieved the highest Pearson correlation (*r* = 0.76) and the lowest MSE (0.55), while SRTsim, scDesign3, and sCCIgen without CCI showed substantially lower correlations (*r* = 0.57, − 0.25, and − 0.14) and higher MSEs (0.82, 1.97, and 1.5), respectively. Similarly, for gene–gene interactions between neighboring cells (Fig. 3 d), sCCIgen with estimated CCIs again outperformed other methods, yielding the highest correlation (*r* = 0.88) and lowest MSE (0.31). In contrast, SRTsim, scDesign3, and sCCIgen without CCI had lower correlations (*r* = 0.1, 0.14, and 0.71) and higher MSEs (1.14, 1.19, and 0.61).

**Fig. 4:**
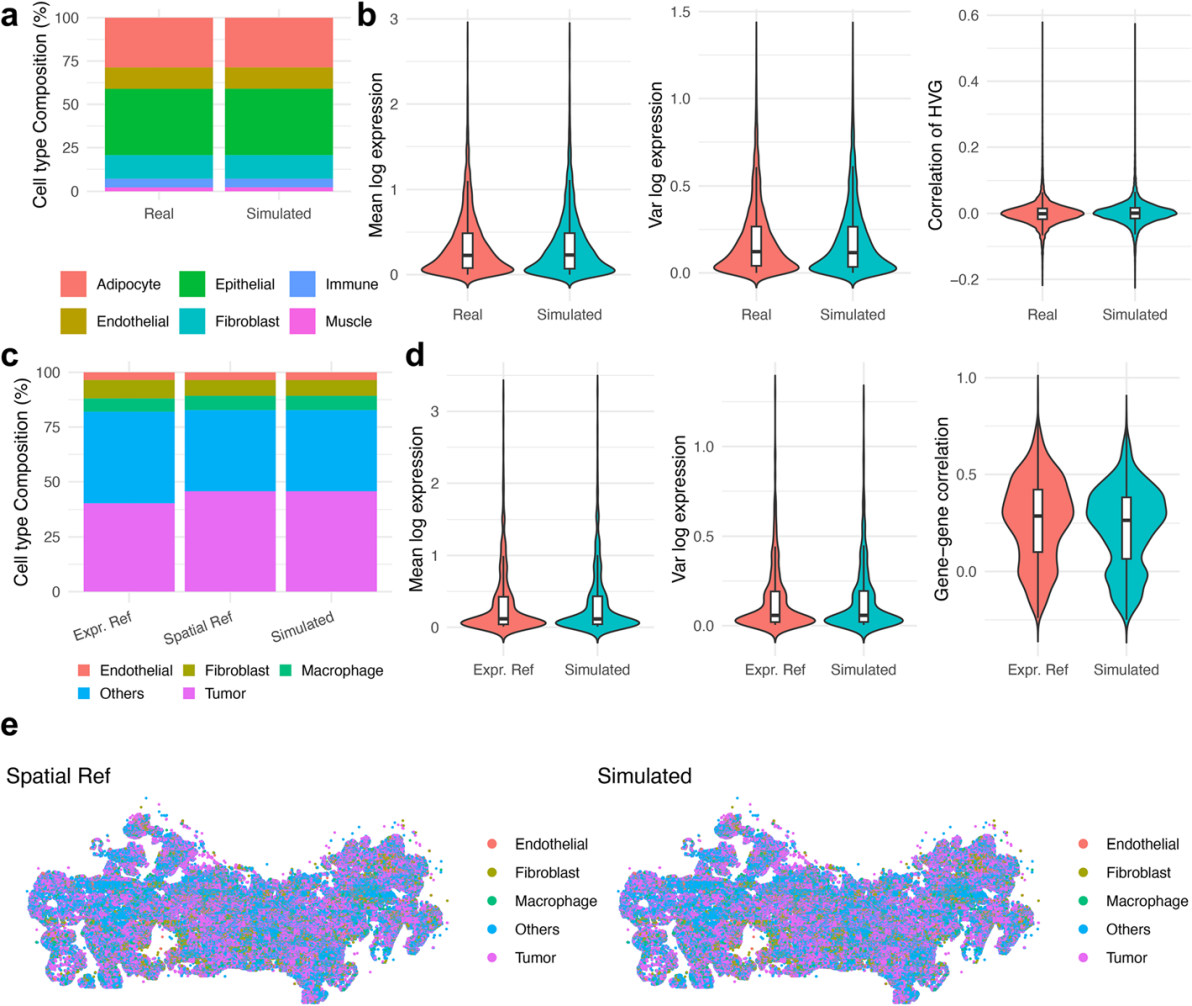
sCCIgen simulated SRT data highly mimics the reference data from snRNAseq and unpaired expression-spatial profiles. **a** Bar plots of cell type composition for six cell types in the reference and simulated data based on snRNAseq of the GTEx breast tissue. **b** Violin plots comparing transcriptomics features between the reference and simulated data, including mean and variance of the log-transformed counts and gene–gene correlations among highly variable genes. **c** Bar plots of the cell type composition for he five cell types across the expression reference, spatial reference, and simulated data based on unpaired ovarian cancer MERFISH reference data. **d** Violin plots comparing transcriptomics features between the reference expression and simulated data, including mean and variance of the log-transformed counts, and gene–gene correlations among highly variable genes. **e** Spatial maps comparing the spatial reference and simulated data

**Fig. 5:**
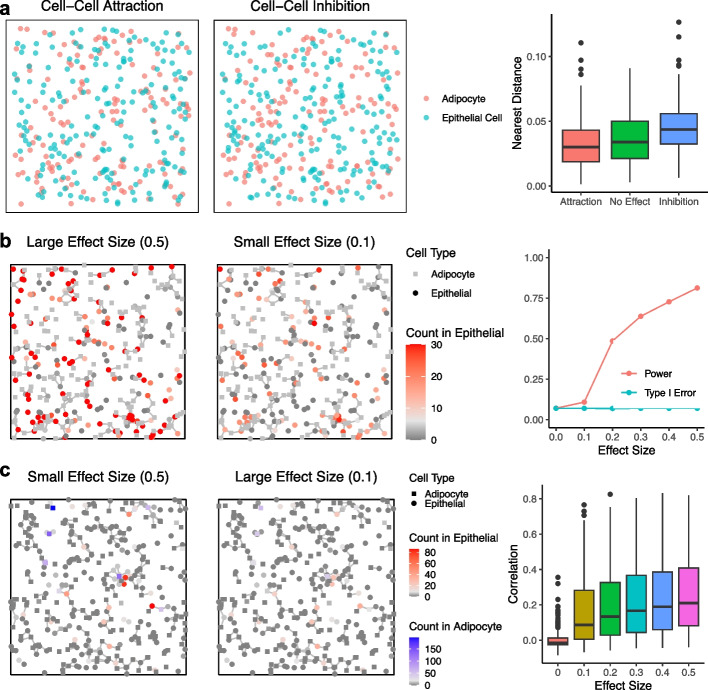
Demonstration of the de novo CCI patterns simulated by sCCIgen based on snRNAseq data of human breast. **a** The spatial maps and boxplots of the simulated cells under the cell–cell location attraction/inhibition patterns. The adipocytes and epithelial cells attract (left) or inhibit (middle) each other from locating in the neighborhood. The boxplots (right) capture the distance from each adipocyte to its nearest epithelial cell under cell–cell attraction, no effect, and cell–cell inhibition. **b** The spatial maps and statistics of the simulated cells under cell–cell spatial dependence of gene expression. The expression of the demonstrated gene (red-gray) in epithelial cells (circles) is simulated to associate with the existence of nearby adipocytes (squares). An edge represents that two cells are nearby and interact. The effect size is the mean shift on the log-transformed read counts for interacting cells. Type I error and power are reported for identifying the CCI-changed genes at different effect sizes. **c** The spatial maps and statistics of the simulated cells under gene–gene interactions between neighboring cells. The expression of the demonstrated gene (red-gray) in epithelial cells (circles) is simulated to associate with the demonstrated gene (blue-gray) in adipocytes (squares). An edge represents that two cells are nearby and interact. The effect size is the mean shift on the log-transformed read counts of both genes in the interacting cells. The boxplots capture the correlations of these interacting genes, with colors indicating different effect sizes

**Fig. 6:**
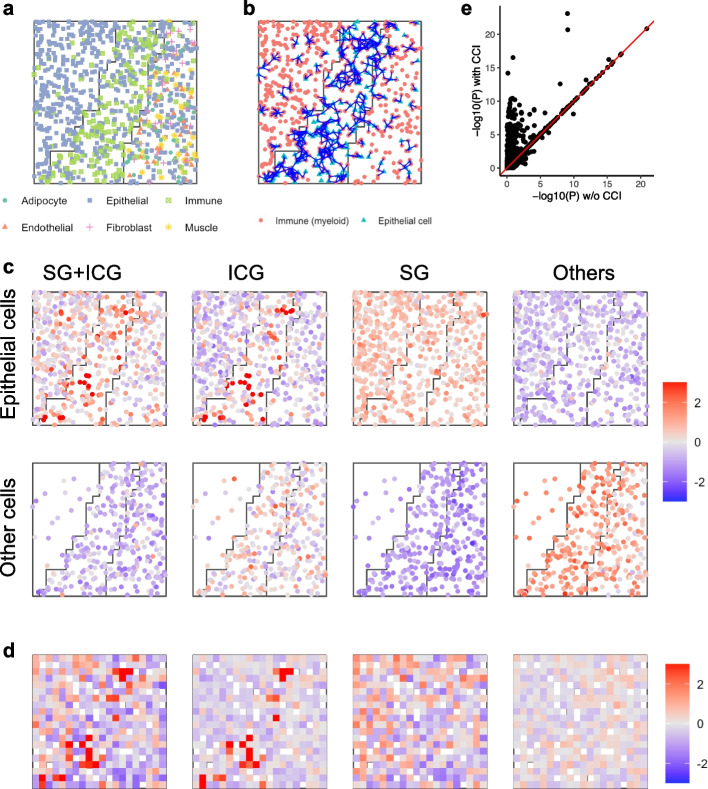
Demonstration of the sCCIgen simulated compounding effects of CCI and spatial variation on cell-type composition. **a** The spatial map of the simulated cells of six cell types in three regions. **b** Illustration of CCIs between epithelial and immune cells on the spatial map. An edge (blue) represents that two cells are nearby and interact. **c** Averaged expression levels in epithelial and other cell types for simulated genes divided into four exclusive groups: epithelial signature gene (SG) + interaction-changed genes (ICG), ICG only, SG only, and others. **d** Averaged expression levels at the multi-cell resolution for the four groups of simulated genes. **e** *P*-values from spatial variation analysis for all genes in simulated data versus w/o epithelial-immune cell–cell interaction

**Fig. 7:**
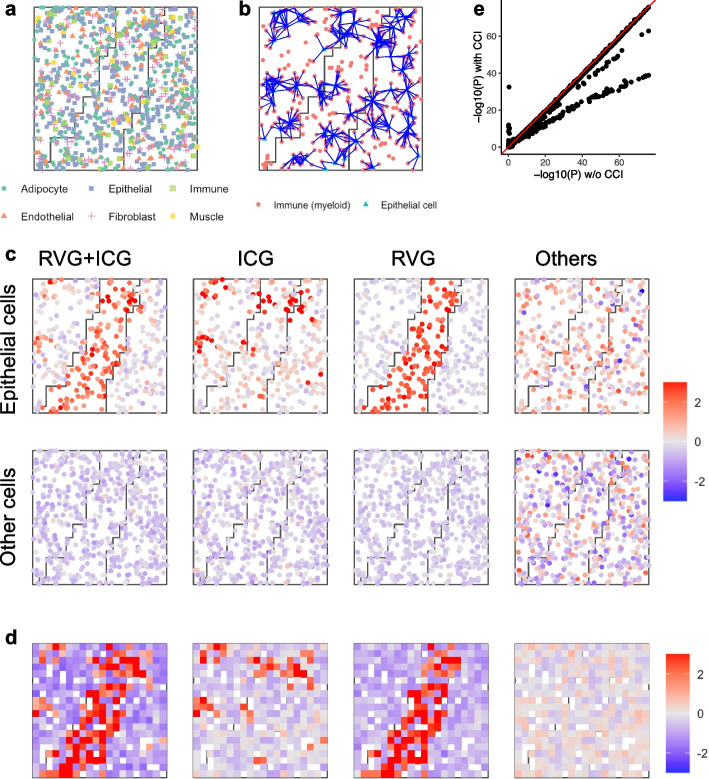
Demonstration of the sCCIgen simulated compounding effects of CCI and cell-type-specific regional differential expression. **a** The spatial map of the simulated cells of six cell types. **b** Illustration of CCIs between epithelial and immune cells on the spatial map. An edge (blue) represents that two cells are nearby and interact. **c** Averaged expression levels in epithelial and other cell types for simulated genes divided into four exclusive groups: regional variation gene (RVG) + ICG, ICG only, RVG only, and others. **d** Averaged expression levels at the multi-cell resolution for the four groups of simulated genes. **e** *P*-values from spatial variation analysis for all genes in simulated data under epithelial-immune CCI versus without CCI

Next, to evaluate sCCIgen under the partial or unpaired reference data, we simulated SRT datasets under two settings: one using snRNAseq without spatial coordinates (Fig. 4a–b), and another using MERFISH data with expression and spatial information from different slides (Fig. 4c–e). The snRNAseq reference was obtained from the mammary tissue of a woman of European Ancestry in the Genotype-Tissue Expression (GTEx) project [21], consisting of 4751 genes from 5990 cells of six cell types, including epithelial cells, adipocytes, fibroblasts, endothelial cells, immune cells, and others. In the simulation, sCCIgen preserved the cell type composition across six annotated cell types (Fig. 4a) and faithfully recapitulated key transcriptomic properties, including mean and variance of log-transformed expression and gene–gene correlations among highly variable genes (Fig. 4b). The spatial patterns were generated de novo as demonstrated in Figs. 5, 6, and 7. The MERFISH reference from an ovarian cancer sample included expression data of 550 genes from 248,065 cells from one slide and a spatial Map of 209,173 cells from a different slide. Cells were annotated into five major types, including tumor (epithelial) cells, fibroblasts, endothelial cells, macrophages, and others. In this simulation, sCCIgen inherited the spatial reference, preserving its cell-type composition (Fig. 4c) and spatial coordinates (Fig. 4e), although it could alternatively generate new cells as demonstrated in Fig. 2. For these cells, sCCIgen simulated their transcriptomic profiles by closely mimicking the transcriptomic features of the expression reference data (Fig. 4 d).

### sCCIgen introduces various de novo CCI patterns

To support the study of CCIs, sCCIgen is also designed to generate de novo patterns of CCI, including cell–cell colocalizations, spatial dependence of gene expression, and gene–gene interactions between neighboring cells. To illustrate this, we simulated SRT datasets referencing the snRNAseq data (Fig. 5). First, to demonstrate the cell–cell colocalization, we simulated different SRT datasets by altering the adipocyte-epithelial cell–cell colocalization parameter. The simulated adipocytes and epithelial cells were placed on the spatial map as if they attract (Fig. 5a; left) or inhibit (Fig. 5a; middle) each other spatially. Box plots capturing cell–cell distance from each adipocyte to its nearest epithelial cell demonstrated their deviations from no CCI (Fig. 5a; right). Next, to demonstrate the spatial dependence of gene expression, we simulated that 10% of the genes in the epithelial cells were positively perturbed by the presence of nearby adipocytes. The expression level of an interacting gene in epithelial cells, at different perturbation effect sizes, together with spatial coordinates with their nearby versus distant adipocytes, was demonstrated (Fig. 5b; left-middle). The larger the effect size, the greater the expression levels were observed in the interacting cells, which were cells with edges to nearby adipocytes, while the expression levels in non-interacting cells with no edges remained the same. Such data could be used for understanding study powers, as well as type I error rates, under different effect sizes for existing analytical tools (Fig. 5b; right). Finally, to demonstrate the gene–gene interactions between neighboring cells, we simulated 10% of genes in adipocytes and the same number of genes in epithelial cells, paired with each other and co-expressed in the nearby interacting adipocyte-epithelial cell pairs. The expression levels of an illustrating gene pair in these two cell types, at different effect sizes, were shown (Fig. 5c; left-middle), together with Pearson correlations of these 10% co-expressed gene pairs at different effect sizes (Fig. 5c; right). The larger the effect size, the greater the expression levels for the pair of genes were observed in the interacting cells, while the expression levels remained the same in non-interacting cells.

### Application of sCCIgen for examining CCI impacts on spatial variation studies

Beyond mimicking the reference data and allowing de novo CCIs, sCCIgen also builds in features to simulate de novo spatial patterns, facilitating the examination of CCI impacts on spatial variation studies. sCCIgen introduces spatial patterns in two ways. First, it allows cell-type composition to vary in different regions, placing cells within distinct microenvironments. Second, it allows certain genes in some cell types to be activated or suppressed in certain spatial regions, such as in response to pathological triggers. Even when cells respond similarly to signals from nearby cells, these spatial patterns can influence their behavior. By building in features to simulate both CCIs and spatial variations, sCCIgen captures the complexity of cellular behaviors under their intricate interplay in real-world data.

To examine the impacts of CCIs on studying spatial variations introduced through varying cell-type composition, we simulated a dataset with three spatial regions of diverse cell-type composition patterns (Fig. 6a). The reference data was again the snRNAseq data from mammary tissue. Across all three regions, the epithelial cells were generated to interact with their neighboring immune cells if they were located close to each other, which is represented by an edge in Fig. 6b. However, the frequency of interactions varied according to the differing densities of epithelial and immune cells, with interactions most frequent in the middle region where the two cell types intermixed the most (Fig. 6b). These randomly simulated interactions perturbed 10% genes, termed interaction-change genes (ICGs), with an increased expression. The normalized averaged expression levels of these ICGs, as well as the epithelial signature genes (SGs), in each epithelial and non-epithelial cell, are presented (Fig. 6c). The epithelial SGs were identified in the reference snRNAseq data from one-sided differential expression analysis comparing epithelial cells with all other cell types combined at a 10% false discovery rate (FDR). Clearly, ICGs were higher in simulated cells in the middle region than in others, epithelial SGs were higher in simulated epithelial cells than in other cells, and their overlapping genes were especially elevated in the simulated epithelial cells of the middle region. The multi-cell resolution data presented the same pattern with enhanced noises (Fig. 6 d). Spatial variable gene (SVG) analysis under cell–cell interactions versus no interactions demonstrated that CCIs drove many genes to become SVGs (Fig. 6e), underscoring the importance of considering CCIs in the analysis and interpretation of spatial variable genes.

Next, we similarly evaluated the impacts of CCI on spatial variations introduced through perturbing expressions in different regions of the same cell type. We simulated the same three spatial regions as before, but this time, different cell types were positioned in the same way in these regions (Fig. 7a). As a result, the simulated epithelial-immune CCIs occurred in similar frequencies in different regions (Fig. 7b). In addition to the interactions, we also introduced spatial variation by increasing the expression levels for 20% of genes in epithelial cells in the middle region, designating this special case of spatial variable genes as regional variable genes (RVGs). The normalized averaged expression levels of these RVGs and ICGs were presented in epithelial and non-epithelial cells (Fig. 7c), demonstrating that epithelial RVGs had higher expression levels in the middle region than other regions and ICGs exhibited clustered elevated levels but no regional patterns, which were consistent with the multi-cell resolution data (Fig. 7 d). Spatial variable gene analysis prioritized different genes under the existence of these CCIs versus no interactions (Fig. 7e), again suggesting that CCIs impact the identification of spatial variable genes.

### Application of sCCIgen for benchmarking scRNAseq/snRNAseq-based LR inference tools

**Fig. 8:**
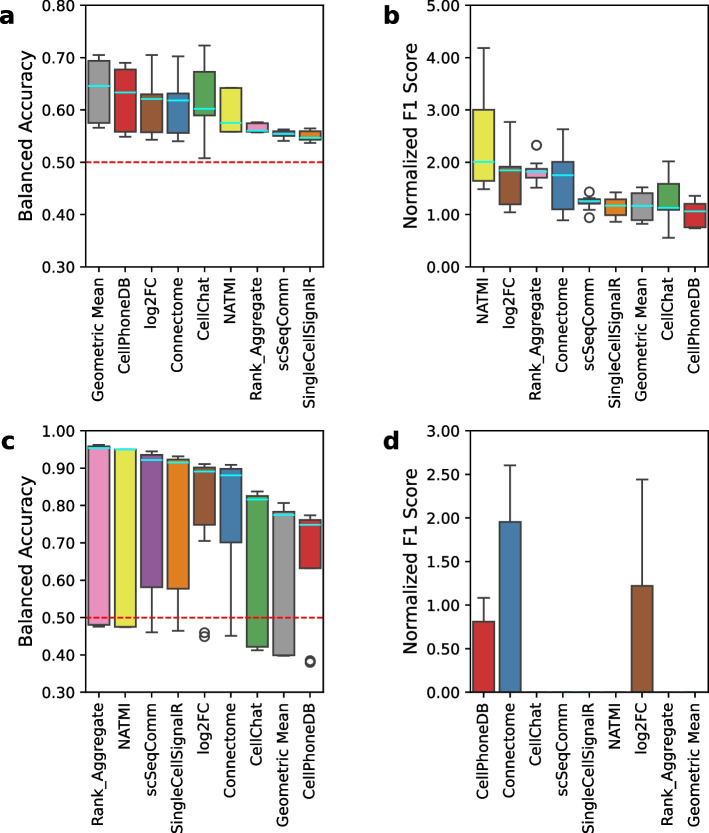
Benchmark scRNAseq/snRNAseq-based LR inference tools using sCCIgen simulated data. The simulation used mouse cortex SeqFISH + data as the reference and generated the ground truth of LR interactions using the estimated effect sizes. Box colors represent LR inference methods. **a**,** b** Balanced accuracy and normalized F1 score for LR inference methods using ground-truth CCI patterns simulated from sCCIgen. **c**,** d** Balanced accuracy and normalized F1 score for LR inference methods against LIANA + -estimated interaction sets from spatial data

To demonstrate the utility of sCCIgen in benchmarking CCI analyses, we simulated 10 independent datasets based on the mouse cortex SeqFISH + data and used them to evaluate the performance of nine ligand-receptor (LR) inference tools derived for scRNAseq/snRNAseq data using a pipeline created by the developers of LIANA + [22]. Each simulated dataset incorporated the LR interactions estimated from the reference data using Giotto [17] in the data generation, providing the ground-truth for rigorous evaluation and comparison of methods. Using balanced accuracy as the evaluation metric, Geometric Mean and CellPhoneDB ranked highest (Fig. 8a), whereas NATMI and log2FC had the highest normalized F1 scores (Fig. 8b). The discrepancy in ranking using these metrics is likely due to their respective weighting of false positive vs false negative errors.

For comparison, we also applied the original LIANA + workflow to evaluate the performance of these LR inference tools, which assumed the ground truth is unknown and used an estimated interaction set for benchmarking. We observed that the ranking of these methods is significantly different when benchmarked against the LIANA + -estimated interaction sets as compared to the ground truth simulated by sCCIgen. In particular, both Genomic Mean and CellPhoneDB performed poorly using the original LIANA + workflow according to the balance accuracy metric (Fig. 8c), whereas NATMI ranked poorly in terms of normalized F1 score (Fig. 8 d). In addition, when using the estimated interaction set instead of ground truth as the benchmark, balanced accuracy varied widely across the 10 simulations, with the first quantile of four methods failing below the random guess threshold (red line in Fig. 8c). Likewise, six out of nine methods yielded a normalized F1 score of zero, due to the absence of overlap between the interactions identified by these LR inference tools and the LIANA + -estimated interaction set, indicating performance worse than random guessing (Fig. 8 d). Taken together, these findings highlight the importance of using rigorously defined ground-truth, as opposed to heuristic estimates, in benchmarking analysis to ensure accuracy as well as robustness.

## Discussion

The advent of SRT technologies has provided researchers with a powerful tool to dissect the intricate landscape of CCIs within the native tissue context. In this manuscript, we introduced sCCIgen, a novel SRT data simulator designed to address the critical need for simulating high-fidelity SRT data with CCIs. sCCIgen is not only able to take SRT as reference data but also to leverage data with only expression profiles (e.g., scRNAseq/snRNAseq) and unpaired spatial and expression profiles from different sources. Its simulated data can faithfully replicate the spatial and transcriptomic features of the reference, capture the CCIs with ground truth, and also add diverse de novo CCI and spatial patterns. It enables the examination of various tools under complex CCIs and spatial variations. sCCIgen offers an R package with a user-friendly interface that includes detailed documentation, tutorials, examples, and pre-simulated data, ensuring ease of use for researchers with varying levels of computational expertise.

Compared to existing simulators such as SRTsim and scDesign3, which we evaluated in this study, and the recently developed scMultiSim [23], which supports multi-omic and spatial simulations, sCCIgen offers a unique focus on the impact of CCI. It enables simultaneous simulation of diverse CCI patterns on cell colocalization, spatial dependence of gene expression, and gene–gene interactions between neighboring cells. It allows variations in simulating these CCI patterns. For instance, as highlighted by CellNeighborEx [24], cell-contact-dependent gene expression changes typically occur over short distances, while ligand-receptor–mediated interactions act over longer ranges. sCCIgen accommodates this by allowing users to specify interaction distances, assigning distinct effects to different cell types and genes. More importantly, sCCIgen largely expanded the applicability of the reference data for SRT simulation: while all existing methods require SRT data for real-data-based simulations, sCCIgen allows data with only expression profiles (e.g., scRNAseq/snRNAseq) and unpaired spatial and expression profiles from different sources. Meanwhile, sDesign3 and scMultiSim are multi-purpose simulators (e.g., simulate chromatin accessibility) with SRT being only one of multiple data types they simulate.

sCCIgen enables diverse applications in spatial transcriptomics experiments, data analyses, and method development. First, it can be naturally applied to study design and power analysis [25] by simulating SRT data under varying expression, spatial patterns, and interaction strengths. Given the complexity of SRT studies, closed-form power calculations are infeasible, making simulation the primary strategy. sCCIgen aids feasibility assessments by estimating the power of observing a signal given available samples and effect size, or conversely, the minimal sample size required to achieve a targeted power. Secondly, sCCIgen provides a flexible platform to benchmark computational methods—such as CCI inference tools, spatial clustering algorithms, and spatially variable gene detection—under controlled ground-truth settings. Our analysis has shown that CCIs can influence the detection of spatially variable genes and that CCI tools perform differently on simulated data. Such analysis can enable refinement of analytical methods and deeper insights into spatially resolved transcriptomic data. More extensive benchmarking, with a wider array of tools, diverse simulations, and systematic evaluation, is beyond the scope of this work but an important direction for future research.

sCCIgen is computationally efficient and scalable to larger experiments. For example, based on the MERFISH reference of 248,065 cells and 550 genes, sCCIgen simulates the expression and spatial profiles for 10,000 cells within 3 min on a single local computer (Apple M3 Max, 48 GB RAM), which includes about 1.9 min on parameter estimation that can be avoided in repetitive simulations. This high efficiency is achieved by building several sampling schemes in sCCIgen for referencing large datasets. Briefly, sCCIgen samples a representative subset of cells (e.g. 2500 per cell type per region) for model fitting, leveraging diminishing returns in accuracy with larger sample sizes. Additionally, it separates model fitting from data generation, enabling faster-repeated simulations from the same reference.

Since sCCIgen is designed with a focus on cell–cell interactions, it is limited to input data at the cellular resolution. Future studies could expand its capabilities by integrating sCCIgen with emerging cell–cell interaction tools that operate at multi-cell or subcellular resolutions, allowing it to process information at different scales. Additionally, sCCIgen relies on pre-annotated cell types and is constrained by their accuracy. For example, if the input data contain cell subtypes that are not labeled, the simulation will treat all cells of that type as homogeneous, potentially obscuring important biological heterogeneity. Therefore, accurate and biologically relevant cell type annotation is crucial to ensure realistic and meaningful simulations. Lastly, sCCIgen is primarily centered around transcriptomics data, while spatial genomics and proteomics data are becoming increasingly available. The count model built into sCCIgen may not be directly applicable to these additional layers of datasets. Extending the statistical models to better describe these additional omics data would enhance sCCIgen’s utility for exploring complex spatial biology.

## Conclusions

sCCIgen provides a flexible, high-fidelity, and user-friendly simulator for spatially resolved transcriptomics that uniquely incorporates cell–cell interactions into synthetic data generation. By leveraging diverse sources of reference data, including paired SRT, single-cell transcriptomics, and ancillary spatial maps, sCCIgen faithfully reproduces transcriptomic and spatial features while enabling the creation of de novo patterns beyond what is captured in existing datasets. This dual capacity strengthens benchmarking and empowers experimental design, offering researchers a powerful platform to test computational tools, evaluate statistical power, and explore biological hypotheses under controlled yet realistic scenarios. As spatial omics technologies continue to evolve, sCCIgen fills a critical methodological gap and will serve as an essential resource for advancing analytical innovation and deepening our understanding of cellular communication in complex tissues.

## Methods

### Overview of the sCCIgen workflow

sCCIgen contains two Major stages. Stage 1 simulates the spatial map (Table 2) and Stage 2 simulates the gene expression profile (Table 3). Based on the three different scenarios, including paired spatial and expression, expression alone, and unpaired spatial and expression, these two stages use different algorithms, but their goals are similar: Stage 1 begins by determining the spatial window in which cells are placed and the regions where they can exhibit distinct profiles. Next, cells are allocated within each region and assigned spatial coordinates. If CCI effects are simulated, sCCIgen either estimates them from data or incorporates user-defined patterns, generates a spatial Map of a large cell pool, or selects a subset that satisfies the specified interactions. Stage 2 first fits the transcriptomics data, and then generates new expression profiles based on fitted models. To fit the data, sCCIgen models the (region-specific) cell-type-specific marginal expression distributions, gene–gene correlations (optional), and CCI effects on spatial dependence of gene expression and gene–gene interactions between neighboring cells (optional, if CCIs are to be simulated based on reference). To generate new data, sCCIgen first predicts the initial counts based on the marginal distributions and gene–gene correlations, next calculates the cumulative changes from the initial counts based on the user-specified CCI and regional variations, and finally updates the counts to capture these effects. Note, when the number of cells in the reference data is large, sCCIgen allows for choosing a subset of cells (per cell type) for modeling to improve computational efficiency. This feature makes sCCIgen scalable and well-suited for large-scale experiments.

**Table 2:** Roadmap of Stage 1 for simulating the spatial map of the cells

	**No Spatial Ref**	**Spatial Ref** **(paired)**	**Spatial Ref** **(unpaired)**
1. Spatial window/region determination	1.1 Random-walk-based algorithm	1.1 Delaunay triangulation network-based algorithm	
2. Initial cell allocation	2.1 Random allocation	2.1 Fit Poisson point process models and predict from models with M-H algorithm	
	2.2 Deletion of cells that are overlapping on spatial map		
3. With CCI incorporation	3.1 Specify de novo cell–cell colocalizations (e.g., cell type pairs and effect sizes)	3.1a Estimate cell–cell colocalization parameters from reference, and/or 3.1b Specify de novo cell–cell colocalization patterns	3.1 Specify de novo cell–cell colocalization patterns
	3.2 Cell number inflation to create pools of cells for selection		
	3.3 Simulate spatial coordinates for all cells in the pool based on 2		
	3.4 Calculate selection probabilities based on: • Attraction and inhibition of the same cell type •Attraction and inhibition of different cell types • Even distribution requirement of cells		
	3.5 Select cells from the pool based on selection probabilities and save the spatial coordinates of these simulated cells

**Table 3:** Roadmap of Stage 2 for generating read counts for cells based on reference data

	**No Spatial Ref**	**Spatial Ref** **(paired)**	**Spatial Ref ** **(unpaired)**
1. Fit the expression reference data		1.1 By region: region-specific model fitting if specified	
	1.2 By cell type: marginal distribution of genes and gene–gene correlation		
		1.3 By cell type pair for CCI: estimate spatial dependence of gene expression and gene–gene interactions between neighboring cells	
2. Generate initial read counts based on fitted (region- and) cell-type-specific models			
3. Calculate the cumulative changes on log read count based on		3.1 estimated CCIs from 1.3	
	3.2 de novo CCIs if specified		
	3.3 de novo regional variations if specified		
4. Update the read counts to capture CCI and regional variations			

### sCCIgen Stage 1: Simulate the spatial map for cells

sCCIgen begins by simulating a spatial map of cells, leveraging available spatial information from the reference data. If no spatial reference is available (e.g., snRNAseq/scRNAseq), users can specify parameters to generate spatial Maps. When spatial data are available, users May either use the existing maps and proceed directly to Step 2 or simulate new cells. In the latter case—whether using the same source as the expression reference or an ancillary source—sCCIgen learns simulation parameters from the data, with the ancillary reference required to share the same cell types as the expression reference.

### User-specified spatial input

When spatial reference is lacking, users specify parameters to generate spatial data, including the number of regions, region-level cell counts, cell-type proportions, and optionally de novo cell–cell colocalizations with defined effect sizes. When spatial information is available (e.g., MERFISH or SeqFISH +), sCCIgen incorporates annotated cell types, cell coordinates (x, y), and regions (if region-level simulation is desired) as input. Users may further adjust cell-type proportions per region, and specify CCI impacts on cell–cell colocalization with the involved cell types and effect size, where reference-based parameters can be estimated internally within sCCIgen. Additional parameters, such as cell density smoothness and overlap removal, can also be set to better mimic real SRT data.

### Random-walk-based algorithm to determine spatial regions when spatial reference is not available

**Fig. 9:**
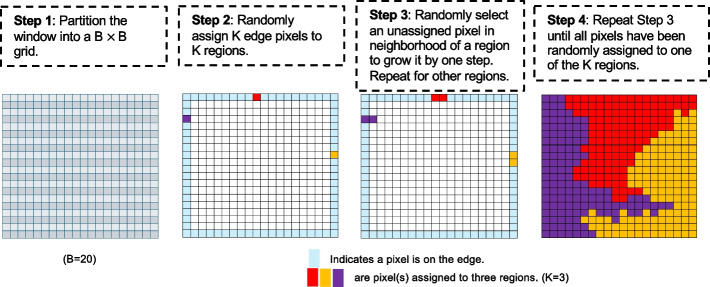
Illustration of random-walk-based algorithm for separating a spatial window into *K* regions

When no spatial reference is available, sCCIgen defines a unit square as the spatial window. If multiple regions (*K* > 1) are specified, it employs a random-walk–based algorithm to partition the window into *K* connected spatial regions (Fig. 9):

- *Step 1. Grid partition. *The window is divided into a *B* × *B* grid, where *B* controls the smoothness of region boundaries (default *B* = *20*). Each grid square is treated as a pixel.
- *Step 2. Initialization*. sCCIgen first identifies border pixels by calculating the number of neighbors for each pixel, where neighbors are defined as edge-sharing pixels. Interior pixels have four neighbors, while border pixels have fewer than four. From these border pixels, sCCIgen randomly selects *K* as the starting points for the *K* regions.
- *Step 3. Region growth*. For all pixels already assigned to a region, sCCIgen identifies all unassigned edge-sharing neighbors and randomly selects one to expand the region.
- *Step 4. Stopping rule*. sCCIgen executes Step 3 sequentially across all *K* regions, expanding each by one pixel per iteration. It grows Region 1 by one pixel, then Region 2, continuing through Region K, and then cycles back to Region 1. Growth for a region stops once all its neighboring pixels are assigned, and the algorithm terminates when all pixels have been allocated.

This algorithm assumes equal-sized regions and partitions the spatial window into *K* randomly connected regions of similar sizes.

### Delaunay triangulation network-based algorithm to determine spatial window and regions when spatial reference is available

When spatial reference is available, sCCIgen develops a novel Delaunay triangulation [26] network-based algorithm to accurately estimate the spatial window for input spatial data with complex shapes.

- *Step 1: Add a circular buffer around cells*. Spatial maps of existing cells typically provide the coordinates of cell centers. To ensure cells are fully contained within the spatial window rather than positioned directly on its boundary, sCCIgen generates a small circular buffer around each cell (e.g., radius = 0.0005), represented by four additional (x, y) points. Without this step, boundary cells may be excluded.
- *Step 2: Build the Delaunay triangulation network*. sCCIgen treats each cell coordinate as a node and identifies spatially adjacent cells by developing edges using Delaunay triangulation. The Delaunay triangulation ensures that no cell lies inside the circumcircle of any triangle formed by other neighboring cells, thereby capturing local spatial neighborhoods without requiring nuisance parameters such as *k*-nearest neighbors or distance thresholds.
- *Step 3: Identify the outer frame as the window*. Before extracting the outer frame, sCCIgen first identifies and deletes the outlier edges of the network. The outlier edges are defined as those exceeding the mean edge length by more than three standard deviations (e.g., > mean + 3SD). With the outlier-excluded network, sCCIgen extracts the outer frame of the network as the window of the spatial data.
- *Step 4 (optional): Determine windows for spatial regions. *The same algorithm can be applied to each spatial region separately. Small overlaps between regions, introduced by buffer extension, are randomly assigned to one region to avoid conflicts.

This four-step algorithm provides an accurate, data-driven estimation of spatial windows and regions. In addition, sCCIgen also supports faster approximation using simpler functions, such as “rectangular” and “convex” polygons, as in existing simulators. It can also split the data into 2–5 sections, estimate the “convex” polygons in each section, and merge the polygons to estimate the window, which balances between accuracy and complexity.

### Initial cell allocation within the spatial window

If no spatial input is provided, cells of each cell type are randomly generated on the spatial map within the simulated regions. If spatial reference is provided, within the estimated spatial window, sCCIgen fits a parametric Poisson point process model [27] to approximate the logarithm density of cells for each cell type as a function of the spatial coordinates $x,y$. For a cell type with more than ten cells, the model considers spatial coordinates $x,y$ with degree $\le 3$ (1, x, y, x^2, y^2, xy, x^3, x^2y, xy^2, and y^3). Specifically, let ${\lambda }_{c}(x,y)$ be the intensity function at the location $(x, y)$ for the cell type $c$, where $c \in (1, \dots , C)$ for $C$ cell types. Then, the Poisson point process model for each cell type $c$ is defined as $log {\lambda }_{c}\left(x, y\right)={\beta }_{0c}+{\beta }_{1c} x+{\beta }_{2c} y+{\beta }_{3c} {x}^{2}+{\beta }_{4c} {y}^{2}+{\beta }_{5c} xy+{\beta }_{6c} {x}^{3}+{\beta }_{7c} {x}^{2}y+{\beta }_{8c}{xy}^{2}+ {\beta }_{9c} {y}^{3}.$

When the number of cells for a cell type is small (e.g., < 10), this model may overfit the data, and we reduce it to consider coordinates $x,y$ with degree $\le 2$ (1, x, y, x^2, y^2, and xy), such that $log {\lambda }_{c}\left(x, y\right)={\beta }_{0c}+{\beta }_{1c} x+{\beta }_{2c} y+{\beta }_{3c} {x}^{2}+{\beta }_{4c} {y}^{2}+{\beta }_{5c} xy.$

Next, to simulate the spatial maps for the user-specified numbers of cells for each cell type, sCCIgen uses the Metropolis–Hastings (M-H) algorithm [28] to predict the spatial coordinates of new cells from the fitted Poisson point process model. Briefly, the M-H algorithm initializes the simulation with a randomly generated cell pattern, then iteratively proposes small modifications to the cell configuration, such as adding or removing cells, and accepts or rejects those proposals based on probabilities determined by the model’s log-linear intensity. For example, in an iteration $i$, a random cell $\left({x}^{*}, {y}^{*}\right)=\left(0.6, 0.2\right)$ is added. The intensity ${\lambda }_{c}^{i}\left({x}^{*}, {y}^{*}\right)$ at this proposed cell can be evaluated, such that $log {\lambda }_{c}^{i}\left({x}^{*}, {y}^{*}\right) ={\widehat{\beta }}_{0c}+{\widehat{\beta }}_{1c} {x}^{*}++{\widehat{\beta }}_{2c} {y}^{*}+{\widehat{\beta }}_{3c} {{x}^{*}}^{2}+{\widehat{\beta }}_{4c} {{y}^{*}}^{2}+{\widehat{\beta }}_{5c} {x}^{*}{y}^{*}+{\widehat{\beta }}_{6c} {x}^{*3}+{\widehat{\beta }}_{7c} {x}^{*2}y+{\widehat{\beta }}_{8c}{x}^{*}{y}^{*2}+ {\widehat{\beta }}_{9c} {y}^{*3}$. sCCIgen accepts this new cell with probability $\min \left(1, \frac{{\lambda }_{c}^{i}\left({x}^{*}, {y}^{*}\right)}{c}\right),$ where $c$ is a scaling factor set to be greater than or equal to the global maximum of density. The M-H algorithm is implemented through the R function *spatstat.random::rmh* with its default setting of 500,000 iterations.

Finally, when the simulated cells are located within the minimally allowed distance, indicating they are overlapping, all but one cell are randomly deleted. Note, if input data covers multiple regions, sCCIgen performs region-level simulation and integrates the spatial map for cells across all regions.

### Cell allocation under the existence of CCI

Under the existence of CCI, sCCIgen first generates a large pool of cells within the spatial regions, and then selects a subset of them based on the specified patterns.

- *Step 1 (optional): Estimate the CCI effects on cell*–*cell attraction and inhibition*.This step is optional, only applicable under the paired spatial-expression reference, and for the purpose of using estimated effects for simulation. sCCIgen first defines cells within vs outside of the neighborhood. sCCIgen employs three strategies for determining neighboring cells: *k*-nearest neighbors, Delaunay triangulation network-based neighbors, and distance-based neighbors. Next, sCCIgen calculates the enrichment score and its corresponding *p*-value through permutation procedures as done in Giotto [17]. Briefly, in each permutation, sCCIgen shuffles the cell type labels of each node (cell), and records the appearance of different cell types in the neighbors. Across large permutations (e.g., 2000), sCCIgen calculates the expected frequency of neighboring cell types by averaging these permutations. Then the enrichment score is defined as the log2 ratio of the observed and expected frequency in the neighbors, and its permutation *p*-value is obtained as the probability of observing as extreme or more extreme scores under the permuted samples.
- *Step 2: Inflate the cell numbers to create a pool of cells to choose from*. The goal of this step is to provide enough cells for selection to generate the spatial map of cells that satisfies the pre-specified patterns. sCCIgen specifies the inflation ratio $\lambda = (\sum_{j}\eta* (1+|a_{j}|))^ {(1 + \nu )},$ where parameter ${a}_{j}$ is the strength of cell–cell inhibition/attraction pattern $j$, which can be estimated from the data; $\nu$ is the cell distribution evenness parameter (default = 0) to be specified by the users; and $\eta$ is the inflation parameter determining the scale of cell number inflation based on CCI and distributional evenness, which is pre-set and can be revised in sCCIgen by matching the signal levels between simulated data and the reference. The stronger the cell–cell attractions/inhibition, the greater the cell distribution evenness, and the larger the inflation parameter are, the bigger the inflated cell numbers will be.
- *Step 3: Generate the spatial coordinates for the inflated number of cells. *sCCIgen generates the spatial coordinates for the entire cell pool using the strategies proposed in the section on “Initial cell allocation within the spatial window.”
- *Step 4: Calculate the selection probability for each cell*. sCCIgen calculates the selection probabilities for cells in each cell type based on (1) attraction and inhibition from the same cell type, (2) attraction and inhibition from different cell types, and (3) the even distribution requirement of cells. It assumes the log odds of selection probability is linearly dependent on the cumulative effects of these parameters. Specifically, for each cell $i$ in a cell type $c$, the logit form of selection probability is defined as

$$logit \left(Pro{b}_{ic}\right)= {\mu }_{1,ic}+ {\mu }_{2,ic}+ {\mu }_{3,ic}+ {\alpha }_{c}.$$

The parameter ${\mu }_{1,ic}$ controls the impact of cell–cell interaction from neighboring cells of the same cell type, ${\mu }_{2,ic}$ controls the impact of cell–cell interaction from neighboring cells of different cell types, ${\mu }_{3,ic}$ controls the cell distribution evenness, and ${\alpha }_{c}$ is a sCCIgen estimated nuisance parameter to reach the overall selection probability.

To calculate $${\mu }_{1,ic}$$, we let $${a}_{j}^{*}$$ be the strength of cell–cell inhibition/attraction pattern $$j$$ for a total of $${J}_{1}$$ interaction patterns among the same cell types as provided from the input of sCCIgen, and $${d}_{i, c_j}$$ be the density of cells in the same cell type $c_j$ in the neighborhood of cell $$i$$ in the initial cell allocation. Then $${\mu }_{1,ic}$$ is the accumulative effects of the product of these two, such that $${\mu }_{1,ic}$$ =$$\sum_{j=1}^{J_1}{a}_{j}{d}_{i, c_j}$$. When cells inhibit each other from occurring in the neighborhood (strength < 0), $${\mu }_{1,ic}<0$$, meaning neighboring cells reduce the selection probability of the cell. When cells attract each other from occurring in the neighborhood (strength > 0), $${\mu }_{1,ic}>0$$, meaning neighboring cells increase the selection probability of the cell.

Similarly, To calculate${\mu }_{2,ic}$, we let ${a}_{j}^{*}$ be the strength of cell–cell inhibition/attraction pattern $j$ for a total of ${J}_{2}$ interaction patterns between different cell types, and ${d}_{i,l_j}$ be the density of cells in the interacting cell type $l$ in the neighborhood of cell $i$ of cell type $c_j$. Then ${\mu }_{2,ic}$ is the accumulative effects of the product of these two, such that ${\mu }_{2,ic}$ = ${\textstyle\sum_{j=1}^{J_2}}a_j^\ast d_{i,l_j}$.

To calculate ${\mu }_{3,ic}$, we let ${d}_{i}^{*}$ denote the density of all cells in the neighborhood of cell $i$ and ${\alpha }^{*}$ is the user-specified cell distribution evenness parameter. Then, ${\mu }_{3,ic} = {d}_{i}^{*} {\alpha }^{*}$ controls the cell distribution evenness.

Finally, ${\alpha }_{c}$ is a nuisance parameter calculated by sCCIgen after ${\mu }_{1,ic}$, ${\mu }_{2,ic}$, and ${\mu }_{3,ic}$ are estimated to ensure the selection probability across all cells matches the ratio between the target and existing cell number in the pool for this cell type.

- Step 5: *Randomly selects cells from the pool based on their selection probabilities. *Based on this selection probability, cells are randomly selected, exhibiting the user-specified spatial patterns. The cell features, including their cell types, spatial coordinates, and regions, are kept as an output to users.

### sCCIgen Stage 2: Simulate expression profiles of the cells

#### Fit the marginal distribution of the genes and their gene–gene correlations

Similar to scDesign2 [29], sCCIgen fits the marginal distribution of the genes and their gene–gene correlations within each cell type. Specifically, for each cell type, sCCIgen fits a zero-inflated negative binomial (ZINB) model for each gene to capture its count data with three parameters: zero proportion, mean count, and overdispersion.

If gene–gene correlation is specified, sCCIgen uses Gaussian copula to model correlation while preserving the marginal distribution of each gene. In detail, let ${Y}_{i}^{c} = {({Y}_{i1}^{c} , {Y}_{i2}^{c} , ..., {Y}_{iG}^{c} )}^{T}$ be the vector of counts for $G$ genes in cell $i$ of cell type $c$, where the marginal distribution of each ${Y}_{ig}^{c}$ follows a ZINB. The joint distribution is defined via a copula to introduce correlation structure as follows:

sCCIgen first introduces a latent Gaussian variable for each gene g, such that ${Z}_{ig}^{c} \sim N (0, 1)$. The joint vector

$${Z}_{i}^{c} = {({Z}_{i1}^{c} , {Z}_{i2}^{c} , ..., {Z}_{iG}^{c} )}^{T}\sim N (0, {\Sigma }_{c}),$$

where ${\Sigma }_{c}$ encodes gene–gene correlations for cell type $c$.

Next, sCCIgen allows 1:1 match of the latent Gaussian variable ${Z}_{ig}^{c}$ with the observed count data ${Y}_{ig}^{c}$ through Gaussian copula transformation, such that

$${U}_{ig}^{c}=\Phi ({Z}_{ig}^{c})$$ and $${Y}_{ig}^{c}={F}_{ZINB, g}^{-1}({U}_{ig}^{c})$$,

where $\Phi$ is the cumulative density function (CDF) of standard normal and ${F}_{ZINB, g}$ is the CDF of the marginal distribution of gene $g$ in $i$. The copula links the marginal ZINB distributions into a joint distribution that preserves the ZINB marginal behavior while allowing flexible, data-driven correlation across genes. The covariance matrix ${\Sigma }_{c}$ (or its correlation version) estimated based on the empirical dependence among genes captures the gene–gene correlations for each cell type.

### Estimate the CCI effects on spatial dependence of gene expression and gene–gene interactions between neighboring cells

Similar to estimating CCI effects on cell–cell attraction and inhibition, this step is optional, applicable only under the existence of paired expression and spatial reference, and requires a determination of neighboring cells. Same as before, sCCIgen allows three strategies for neighboring determination: *k*-nearest neighbors, Delaunay triangulation network-based neighbors, and distance-based neighbors. To estimate the CCI effects on spatial dependence of gene expression, sCCIgen compares cells within vs outside of the neighborhood for each pair of sender-receiver cell types (e.g., T cell –- > B cell), and calculates the enrichment score as log2 fold change of expression level between neighboring and non-neighboring and reports its corresponding permutation *p*-value. To estimate the CCI effects on gene–gene interactions between neighboring cells, due to the large number of potential comparisons (e.g., 400 million tests for 20,000 genes), sCCIgen requires a list of gene pairs to narrow the analysis. To facilitate the application, sCCIgen provides human and mouse ligand-receptor gene lists from CellChat but also allows users to provide their own gene list. Then, sCCIgen computes the enrichment score as the log2 fold change of the product of the first gene in the sender cells and its pairing gene in the receiver cells and reports its corresponding *p*-values for each gene pair in each ordered sender-receiver cell type pair.

### Generate the initial read counts based on the fitted models

For the simulated $n$ cells with spatial coordinates in the spatial maps, sCCIgen simulates their initial read counts based on the estimated marginal distributions of $G$ genes and gene–gene correlations ${\Sigma }_{c}$. Specifically, with users-specified sequencing depth (default = 1; no change), sCCIgen first generates ${n}_{c}\times G$ correlated error terms for each cell type based on the gene–gene correlations ${\Sigma }_{c}$, next it employs the inverse of the Gaussian copula transformation, such that ${U}_{ig}^{c}=\Phi ({Z}_{ig}^{c})$ and ${Y}_{ig}^{c}={F}_{ZINB, g}^{-1}({U}_{ig}^{c})$, to obtain the predicted initial counts based on the models. These predicted counts are cell-type specific and can be region-specific as determined by users’ specifications.

### Calculate the cumulative impacts of CCIs and regional variations on log read counts

sCCIgen allows an update of the expression levels from the initial read counts generated from the reference data due to the CCIs and regional variations. sCCIgen starts by defining a $n\times G$ matrix $\Delta$ with all elements as zero to track the changes from the initial log read counts.

To incorporate the CCI impacts on the spatial dependence of gene expression, users need to provide an input including the interaction region(s) $r$, perturbed cell type(s) $k$, neighbor cell type(s)$l$, interacting distance threshold(s) $c$, perturbed gene(s) $g$, and the mean $\mu$ and standard deviation $\sigma$ of effect size(s). The input can come from estimated values from the data and de novo patterns specified by the users. For de novo patterns, users can require sCCIgen to randomly generate perturbations by specifying the percentage of perturbed genes instead of providing gene names. With this input, elements from the matrix $\Delta$ that corresponds to gene $g$ in the cells that are in region $r$, of cell type $k$, with cell type $l$ within the physical distance $c$, are added with an effect size randomly generated from $N(\mu ,{\sigma }^{2})$.

Similarly, to incorporate the CCI impacts on the gene–gene interactions between neighboring cells, users need to provide an input including the interaction region(s) $r$, perturbed cell type(s) $k$, neighbor cell type(s) $l$, interacting distance threshold(s) $c$, perturbed gene pair(s) $m\to n$, whether the perturbation is bidirectional ($TRUE \text{ vs } FALSE$), and the mean $\mu$ and standard deviation $\sigma$ of effect size(s) of the perturbation. The input can also come from estimated values from the data and de novo patterns specified by the users. With this input, elements from the matrix $\Delta$ that corresponds to gene $m$ in the cells of cell type $k$, in region $r$, with cell type $l$ within the physical distance $c$, are added with an effect size randomly generated from $N(\mu ,{\sigma }^{2})$. If bidirectional is $TRUE$, elements from the matrix $\Delta$ that corresponds to gene $n$ in the cells of cell type $l$, in region $r$, with cell type $k$ within the physical distance $c$, are also added with the same effect size.

Finally, to incorporate de novo spatial variation of gene expression, sCCIgen allows users to specify spatial regions of the input data including the perturbed region(s) $r$, cell type(s) $k$, and gene(s) g, and the mean $\mu$ and standard deviation $\sigma$ of effect size(s) of the perturbation. With this input, elements from the matrix $\Delta$ that corresponds to gene $g$ in the cells that are in region $r$, of cell type $k$, are added with an effect size randomly generated from $N(\mu ,{\sigma }^{2})$.

sCCIgen allows users to provide simultaneous diverse CCI and regional variations, covering different regions, cell types, and genes, with different patterns, and calculate their accumulative effects on log scale by summing up all the effects to create an updated change matrix ${\Delta }^{*}.$

### Update the read counts to capture the CCI and regional variations

The final change matrix ${\Delta }^{*}$ is added to the log-transformed counts to update the read counts. The resulting matrix may have several unfavorable features, and to ensure realistic simulation, sCCIgen builds in a few options to constrain the updated read counts. First, sCCIgen forces minimal counts to be zero, so negative perturbations on genes already with zero counts will not change expression levels in these cells. Second, sCCIgen provides an option to bound the maximum counts of extreme genes to the maximum of these two: (1) five times of the 97.5th quantile level of all genes and (2) the maximum value of the initial read counts. Third, as added CCI and regional variations may alter total counts, sCCIgen provides an option to calculate the ratio of updated vs initial sequencing depth to adjust the counts for all genes and cells to match the sequencing depth. Finally, sCCIgen rounds the final values to the nearest integer to mimic counts.

### sCCIgen multi-cell simulation

When generating multi-cell SRT data, sCCIgen cuts the window into squares of equal size. The number of squares is specified by users and suggested to cover ~ 0–40 cells, mimicking a predetermined SRT spot. The spatial coordinates are the centers of the squares, and the expression levels are summed for all cells in the squares.

### User-friendly interface

To facilitate the usage of sCCIgen, we developed an interactive Shiny application using the packages shiny (version 1.10.0) [30], minUI (version 0.1.2), and shinyFiles (version 0.9.3) [31]. The interactive application is contained in the function run_interactive_sCCIgen(), which launches a local server-free shiny interface. The application contains three main options: (1) download a previously simulated dataset, (2) create a parameter file, or (3) run a simulation. In addition to downloading pre-simulated datasets, the app provides the option of downloading test and real datasets that will be used as the reference for running the simulation. Alternatively, the user can select their own local expression and metadata files as the reference data. When creating a parameter file, the application shows multiple interactive questions that will lead the user through a series of customized options to select the proper parameters and values depending on the dataset of reference used. Finally, we integrated sCCIgen with the Giotto [17] (version 4.2.1), Seurat (version 5.3.0) [32], and SpatialExperiment (version 1.18.1) [19] packages by providing the option of creating and saving a Giotto, Seurat, or SpatialExperiment object that contains the simulated dataset, in addition to the default output files. The integration with these packages will facilitate running downstream spatial analysis with them.

### Data analysis

#### Reference datasets

Three real reference datasets are used for sCCIgen simulation. The SeqFISH + reference dataset was obtained from the cortex of a 23-day-old male mouse (C57BL/6 J) [33]. We used the spatial Maps of 511 cells from five fields of view from the cortex and these cells were categorized by the original study into six major cell types: excitatory neuron (*n* = 325), interneuron (*n* = 42), astrocyte (*n* = 54), endothelial (*n* = 45), oligodendrocyte (*n* = 29), and microglia (*n* = 16). The expression levels of this dataset included 10,000 genes.

The snRNAseq data was from the mammary tissue of one European Ancestry woman “'GTEX-1R9PN” downloaded from the Genotype-Tissue Expression (GTEx) project [34]. The datasets included the expression profile for a total of 4751 genes from 5990 cells of six cell types: epithelial (*n* = 2292), adipocyte (*n* = 1721), fibroblast (*n* = 804), endothelial cell (*n* = 737), immune (*n* = 301), and other (*n* = 135).

The MERFISH ovarian cancer dataset was downloaded from Vizgen [35]. To mimic the unpaired spatial and expression data from different sources, we used the expression profile from slide 1 and the spatial Map from slide 3 of patient 2. The expression data included a total of 550 genes from 248,065 cells of five cell types annotated in a prior study [36], including tumor (epithelial) cells (*n* = 99,572), endothelial cells (*n* = 8,707), fibroblast (*n* = 20,937), macrophages (*n* = 14,984), and others (*n* = 10,385). The spatial reference data included a total of 209,173 cells from the same five cell types, including tumor (epithelial) cells (*n* = 95,481), endothelial cells (*n* = 7,424), fibroblast (*n* = 15,028), macrophages (*n* = 13,659), and others (*n* = 77,581).

### sCCIgen simulation based on reference data

We performed various simulations using sCCIgen based on the three reference datasets. The SeqFISH + based simulations used all 511 cells and 2500 highly variable genes. Prior to simulation, we estimated the spatial and expression features of the data, including gene expression marginal distributions, gene–gene correlations within the same cell types, cell–cell localizations, spatial dependences of gene expression, and gene–gene interactions between neighboring cells. Then, we simulated the spatial Maps of a new 511 cells with and without incorporating CCI impacts on cell–cell localization (Fig. 2). We simulated the expression profiles of these 511 cells using the estimated marginal gene expression distribution and gene–gene correlation, with and without incorporating CCI impacts on spatial dependence of gene expression and gene–gene interactions between neighboring cells (Fig. 3). Additionally, to benchmark scRNAseq/snRNAseq-based LR inference tools, we simulated 10 datasets with 511 cells with all three patterns of estimated CCIs incorporated as input in the simulation.

The snRNAseq based simulations used all 4751 genes from 5990 cells. Due to the lack of spatial reference for this simulation, we estimated the cell-type-specific marginal distributions of gene expression and gene–gene correlations. Then, we simulated the spatial and expression profiles of 5990 new cells without entering de novo patterns (Fig. 4a and b). To demonstrate the de novo CCI patterns, we simulated 500 cells with CCIs impacting the adipocyte-epithelial cell–cell localization (effect size = − 3, 0, 3) (Fig. 5a), the spatial dependences of their gene expressions (effect size = 0, 0.1, …, 0.5) (Fig. 5b), and the gene–gene interactions between their neighboring cells (effect size = 0, 0.1, …, 0.5) (Fig. 5c). To demonstrate the utilities of simultaneously generating CCI and regional variations, we simulated 1000 cells in three regions, first with user-specified different cell type composition in these regions (Fig. 6), and next with regional highly expressed genes in Region 1 for 20% of genes (Fig. 7).

To demonstrate sCCIgen’s capacities for leveraging the unpaired spatial and expression data for simulation, the MERFISH based simulations used the expression profile of slide 1 with 550 genes from 248,065 cells, and the spatial profile of slide 3 with 209,173 cells. We directly used the spatial Map of the 209,173 cells on slide 2 and simulated the expression profile for them (Fig. 4c–e). Similar as before, we estimated the cell-type-specific marginal distributions of gene expression and gene–gene correlations based on expression profile.

### Performance comparison with existing methods

To compare sCCIgen with existing methods, we simulated synthetic SRT data sets from SRTsim, scDesign3, sCCIgen without specifying CCIs, and sCCIgen using estimated CCIs from the reference data based on the same SeqFISH + reference data. To ensure fair comparison, we generated datasets with the same number of cells and genes as in the reference data across all methods.

For SRTsim simulations, we used the SRTsim package and began by fitting a model to the reference expression data using the srtsim_fit() function. The marginal distribution (marginal) was set to its default value, “auto_choose,” allowing the method to automatically select the best-fitting distribution via likelihood ratio tests. The simulation scheme (sim_scheme) was set to “tissue,” indicating a tissue-based simulation approach. Next, we used the srtsim_newlocs() function to generate random spatial coordinates based on the reference locations, with the loc_layout parameter set to “random” to specify a random spatial arrangement from the model. Finally, we applied the srtsim_count() function to generate simulated count data using the estimated expression model and newly generated spatial coordinates. All other parameters were left at their default settings.

For scDesign3 simulation, we used function scdesign3() from the scDesign3 package, and specified its parameter to allow random generation of spatial locations from its models. To model the mean value of gene expression, we used a cell type indicator by setting “celltype” for the parameter “mu_formula.” To model the covariance structure of gene expression, we used value “1” for the formula of variance parameter “sigma_formula” and correlation parameter “corr_formula,” indicating the dispersion parameter is assumed to be constant across all cells. We also set other parameters for expression modeling as instructed from the package, including negative binomial distribution “nb” was used for the marginal distribution “family_use”; and “gaussian” copula was used in the gene expression modeling. For parallel computing, we used 2 cores and set “pbmcapply” as the parallelization function.

For sCCIgen simulation, we first simulated one version without (w/o) specifying any CCIs, and next a second version using three patterns of estimated CCIs from the reference data with its built-in CCI estimation functions. As a result, we have the spatial and expression data from five sources: the reference, SRTsim, scDesign3, sCCIgen w/o CCI, and sCCIgen with est. CCI. For visualization of these data, we plotted their spatial maps for the cells and UMAP (Uniform Manifold Approximation and Projection) for gene expression profiles of highly variable genes in these cells. For quantitative evaluation of the simulated datasets, we compared them to the reference data in term of the spatial window/area, cell-type composition, cell–cell attraction/inhibition pattern, marginal distribution of the gene expression (mean and variance), gene–gene correlation, CCI impacts on cell spatial dependence of gene expression, and CCI impacts on gene–gene interactions between neighboring cells. We calculated the absolute bias (AB) as the difference from the reference data, relative bias (RB) as the difference from the reference data divided by the difference from the reference data, Pearson correlation, and mean squared errors as appropriate.

### Benchmarking the snRNAseq/scRNAseq-based CCI analytical tools

To demonstrate the utility of sCCIgen in benchmarking existing CCI analytical tools, we simulated 10 independent datasets based on the mouse cortex SeqFISH + data, and used them to evaluate nine approaches that identify ligand-receptor (LR) interactions based on snRNAseq/scRNAseq data. Before applying these methods, we normalized the count matrices using total count normalization and log-transformation, and integrated the spatial coordinates into AnnData objects. Then, we used the workflow created by the developers of LIANA + to compare these LR inference methods, including CellPhoneDB[37], Connectome[38], NATMI[39], log2FC-based approaches, SingleCellSignalR, scSeqComm[40], CellChat[41], Geometric mean, and Rank Aggregate. Identifications from each LR inference method were achieved by using method-specific criteria, such as *p*-value thresholds or score cutoffs. To generate the interaction set used as the spatial ground truth according to the LIANA+ pipeline, spatial neighborhood graphs were constructed using a bandwidth of 200 and a cutoff of 0.1. Bivariate Moran’s *I* statistics were computed to characterize spatial patterns of LR pairs and cell-type adjacency. As our simulated data has ground truth, which includes ligand, receptor, source cell type, target cell type, interaction distances, and effect sizes, we also used our custom ground truth for comparison with the LIANA+ ’s spatially estimated interaction set.

The identified LR interactions from these nine approaches were compared to two sets of “ground truth” using two evaluation metrics: the balanced accuracy and normalized F1 scores. Balanced accuracy is calculated by averaging sensitivity (true positive rate) and specificity (true negative rate). It reflects the method’s ability to correctly classify LR pairs while accounting for class imbalance. F1 score represents the harmonic mean of precision and recall and is calculated using sklearn.metrics.f1_score. The normalized F1 score is calculated by taking the ratio of the “observed” F1 score for the actual LR predictions and the “permuted” F1 score generated by shuffling the predictions of each LR method 100 times. Note, when no true positives were identified in the observed data, both F1 score and its normalized score had zero values.

## Data Availability

sCCIgen is publicly available on the GitHub repository https://github.com/songxiaoyu/sCCIgen under an open source GNU General Public License, and Zenodo (DOI: 15,832,711) [42]. A Docker image has also been available at https://hub.docker.com/r/sccigenpackage/sccigen. Tutorials and documentation are available on the sCCIgen website at https://songxiaoyu.github.io/sCCIgen. The pre-simulated data, their reference datasets, analysis codes, and sample parameter files are publicly available at https://github.com/songxiaoyu/sCCIgen_data [43]. The following datasets were used for demonstrations: The SeqFISH + mouse cortex [33] was downloaded from the original paper GitHub repository available at https://github.com/CaiGroup/seqFISH-PLUS. The mammary tissue snRNAseq data 'GTEX-1R9PN' was downloaded from the Genotype-Tissue Expression (GTEx) project [34] and is available at https://www.gtexportal.org/home/downloads/adult-gtex/single_cell. The MERFISH ovarian cancer dataset was downloaded from Vizgen [35] and is available at https://console.cloud.google.com/storage/browser/vz-ffpe-showcase/HumanOvarianCancerPatient1.
